# Re-Evaluation of Cardiovascular Disease Risk and Primary Prevention Treatments with Coronary Artery Calcium Scoring in Primary Prevention Patients

**DOI:** 10.3390/jcm13144125

**Published:** 2024-07-15

**Authors:** Abdulla Arslan, Fatih Aytemiz, İclal Işıklar, Öykü Gülmez Özkaya

**Affiliations:** 1 Department of Cardiology, Baskent University, Istanbul 34662, Turkey; oykug@baskent.edu.tr; 2 Department of Cardiology, City Hospital of Manisa, Istanbul 45030, Turkey; fatih.aytemiz@saglik.gov.tr; 3 Department of Radiology, Baskent University, Istanbul 34662, Turkey; iclal_isiklar@yahoo.com

**Keywords:** atherosclerosis, coronary heart disease, coronary artery disease, hyperlipidemia

## Abstract

**Objective:** The coronary artery calcium score (CACS) is used as a screening tool to identify the presence/absence of subclinical atherosclerosis in asymptomatic individuals. We evaluated the risk categories and medical therapy of asymptomatic individuals with subclinical atherosclerosis (CACS > 0) and applied the atherosclerotic cardiovascular disease (ASCVD) score and Framingham risk score (FRS) to assess those at a high risk of subclinical atherosclerosis (CACS ≥ 400). **Methods:** We retrospectively enrolled 218 asymptomatic individuals (65.6% women, and mean age 67.5 ± 10.3 years) who had their CACS evaluated at the cardiovascular department of our hospital between 2016 and 2020. **Results:** Among the 218 participants, 24.3% were classified as low-risk according to the FRS, and 19.3% had no subclinical atherosclerosis. However, only 12.8% and 27.5% of the study population were taking statins and aspirin, respectively. Furthermore, although more than half of the individuals without subclinical atherosclerosis were in the intermediate- and high-risk groups according to the risk scores, there were no considerable differences in the rates of taking aspirin and statins between the groups. When patients in the very-high-risk group according to the CACS and low-intermediate-risk patients were compared, there was no considerable difference in the rates of risk subgroups and taking statins, whereas high-risk patients took statistically significantly more aspirin. **Conclusions:** In primary prevention screening, CACS can be used as a reliable marker of subclinical ASCVD and help physicians optimize and improve adherence to medical therapy, including aspirin and statins, particularly for high-risk individuals.

## 1. Introduction

Coronary artery disease (CAD) is one of the leading causes of mortality and morbidity worldwide. For the purposes of prevention, primary care physicians must identify the risk status of asymptomatic individuals for developing CAD. Different risk assessment calculators use different parameters, such as age, sex, smoking status, diabetes, hyperlipidemia, blood pressure, family history of premature CAD, and vascular inflammation to estimate cardiovascular (CV) risk [1,2]. Although these risk scoring systems provide a quick and cost-effective method to identify those at increased risk of CV events, many individuals without known risk factors or at low–intermediate risk for CAD still develop CV events [3].

Coronary artery calcium score (CACS) is used in asymptomatic individuals to estimate the presence and burden of coronary atherosclerosis to improve risk stratification and risk reclassification [4]. Although there are different CACS systems, the most commonly used system is the Agatston score, which is the numerical sum of all calcified lesions detected by non-contrast computed tomography. As a result, nonzero calcium scores can be a reliable marker of subclinical atherosclerosis [5]. Several studies have shown the value of CACS in modifying the CV risk prediction in different populations [6,7,8,9,10,11,12]. As one would expect, higher scores are associated with higher CV risk, whereas a zero calcium score is a negative risk marker, indicating a maximum risk of CV disease of 0.5% per year [13]. Moreover, previous studies have shown that CACS can improve the identification of individuals who would benefit from primary prevention medications [14,15]. Although CACS has limited utility in reclassifying the CV risk of low- and high-risk patients, it benefits intermediate-risk patients the most when combined with traditional risk calculators [16,17,18]. Current guidelines recommend performing CACS as a screening tool in asymptomatic individuals without clinical atherosclerotic cardiovascular disease (ASCVD) at the age of 40–75 in the 5%–20% ten-year ASCVD group [19].

The aims of this retrospective study were 2-fold: 1. to evaluate the risk categories and medical therapy of asymptomatic individuals who have subclinical atherosclerosis, defined as a CACS greater than 0; and 2. to assess the risk categories and medical therapy of asymptomatic individuals who are at high risk, defined as a CACS ≥ 400, using the ASCVD score and Framingham risk score (FRS) chart as traditional risk assessment models, retrospectively.

## 2. Materials and Methods

### 2.1. Study Population

A total of 218 individuals above 18 years (65.6% women, and mean age 67.5 ± 10.3 years) who presented for cardiological evaluation at our cardiology department and underwent CACS between 2016 and 2020 were included in this study. Patients with documented CAD, valvular heart disease, peripheral arterial disease, and congenital heart disease at the time of enrolment were excluded. A family history of premature CAD was defined as having a first-degree relative below 60 years of age or a second-degree relative below 50 years of age with CAD. Diabetes was defined as self-reported diabetes or the use of hypoglycemic drugs. Hypertension was defined as untreated blood pressure >140/90 mmHg, or the use of any antihypertensive medication. Dyslipidemia was defined as a prior diagnosis of dyslipidemia or treatment with any lipid-lowering drug. In patients with concomitant laboratory data, dyslipidemia was additionally considered present if low-density cholesterol (LDL-C) >160 mg/dL, high-density cholesterol (HDL-C) <40 mg/dL in men and <50 mg/dL in women, or fasting triglycerides (TG) >150 mg/dL. Smoking status was categorized as never, current, or prior use of cigarettes. Blood samples were taken after 12 h of fasting, and glucose, total cholesterol, high-density lipoprotein, low-density lipoprotein, triglyceride, and hemoglobin A1c values were recorded at the time of the CACS. The FRS and the ASCVD score models were used to determine the risk of incident CV events. Using FRS, all study participants were classified into low-, intermediate-, and high-risk categories with a 10-year coronary heart disease (CHD) risk of ≤10%, 10–20%, and ≥20%, respectively [20]. The 10-year risk of fatal CV disease calculated from the ASCVD score risk chart was grouped as 1–5% for intermediate risk, ≥5% for high risk, and ≥10% for very high risk [21]. The study was conducted in accordance with the Declaration of Helsinki, and approved by the Ethics Committee of Baskent University (approval no. KA21/152 and date of approval 25 March 2021). Signatures for the informed consent form were obtained from the patients, giving their permission.

### 2.2. Coronary Artery Calcium Scoring

CACS was performed using electrocardiographically gated (at 80% of the R–R interval), axial, non-contrast 128-slice chest computed tomographic scans with a 3 mm slice thickness (Siemens Medical Systems, Erlangen, Germany). CAC was manually selected by a trained radiologist. CACS is traditionally categorized as follows: 0 = very low probability of CAD; 1–10 = low probability of CAD; 11–100 = mild or minimal coronary artery stenosis; 101–400 = nonobstructive CAD likely, although obstructive disease is possible; and ≥400 = high likelihood of at least one considerable coronary stenosis [5].

Individuals with a CACS of 0 were grouped as participants without subclinical atherosclerosis (Group 1), whereas individuals with a CACS greater than 0 were grouped as participants with subclinical atherosclerosis (Group 2). Similarly, individuals with a CACS < 400 were grouped as individuals with a low–intermediate risk of CAD (Group A), whereas those with a CACS ≥ 400 were grouped as individuals with a high risk of CAD (Group B).

### 2.3. Statistical Analysis

The statistical package SPSS (SPSS for Windows 16.0, Chicago, IL, USA) was used for statistical analysis. All continuous variables were checked with the Kolmogorov–Smirnov normality test to show their distributions. Continuous variables with normal distributions were compared using the unpaired Student’s *t*-test and are presented as the mean ± SD. For categorical variables, which are reported as frequencies and percentages, the chi-square test was used. Values for *p* < 0.05 were considered statistically significant.

## 3. Results

The demographic and clinical characteristics of the study population are summarized in Table 1. This study included 218 patients with a mean age of 67.5 ± 10.3 years and 75 (34.4%) males. Although the proportion of traditional risk factors, except hypertension (67.9%) and hyperlipidemia (47.7%), was relatively lower in the study cohort, the mean FRS was in the intermediate- or high-risk range, and the mean ASCVD score was in the high-risk range (16.5 ± 9.9 and 9.8 ± 3.3, respectively). The subgroups of the 10-year risk of the study population calculated by the FRS and ASCVD score are also shown in Table 1. Most of the individuals (53.7%) were in the very-high-risk range based on the ASCVD score, whereas 31.2% of individuals were in the high-risk range based on the FRS. Furthermore, 44.5% of the study population was at intermediate risk based on the FRS, and only 7.3% of the study population was at intermediate risk based on the ASCVD score. Conspicuously, in the overall study population, the proportion of baseline medication used for primary prevention was lower among the participants. Although almost half of the individuals were hyperlipidemic, and classified to the high-risk range based on the ASCVD score, only 12.8% were taking statins and 27.5% were taking aspirin (Table 1).

We further divided the patients into two different subgroups based on CACS and studied the distribution of baseline demographic and clinical characteristics of the study population according to these groups. First, we divided the study population into two groups: Group 1 includes individuals with a CACS of 0 as participants without subclinical atherosclerosis, and Group 2 includes individuals with a CACS > 0 as participants with subclinical atherosclerosis. Similarly, Group A included individuals with CACS < 400 as participants with low-intermediate risk of CAD, and Group B included individuals with CACS ≥ 400 as participants with high risk of CAD.

In the overall study population, 42 (19.3%) individuals had a CACS of 0 (Group 1), and 176 (80.7%) individuals had a CACS over 0 (Group 2). Table 2 shows the selected clinical laboratory values and baseline medications of the groups. Age, hypertension, and 10-year FRS were significantly higher in Group 2 (*p* = 0.001, *p* = 0.01 and *p* = 0.04, respectively). Although there was no considerable difference in the 10-year FRS subgroups and 10-year ASCVD score subgroups (*p* > 0.05) between the groups, 26.2% of the individuals were defined as high-risk according to FRS, whereas 31.0% and 54.8% of the individuals were defined as high-risk and very-high-risk according to the ASCVD score in Group 1. When patients were evaluated in terms of the treatment they were receiving, it was determined that the patients in Group 2 were receiving more angiotensin-converting enzyme inhibitors or receptor blockers (*p* = 0.001). Interestingly, the number of patients receiving aspirin and statin therapy in both groups was quite low and there was no statistical difference (21.4% vs. 29% and 14.3% vs. 12.5% respectively; *p* > 0.05). Furthermore, fasting glucose, HbA1c, total cholesterol, LDL, and HDL levels did not differ between the groups (*p* > 0.05).

We further studied the selected clinical laboratory values and baseline medications of Groups A and B (Table 3). In all, 182 (83.5%) individuals had a CACS < 400, while 36 (16.5%) had a CACS ≥ 400. Individuals in Group B were older, had higher fasting glucose and serum creatinine levels, and received more frequent medications, including aspirin and angiotensin-converting enzyme inhibitors or receptor blockers (47.2% and 72.2%, respectively; *p* < 0.05), but only 14% of Group B individuals were taking statins. The mean 10-year FRS and ASCVD score risks were not different between the groups (*p* = 0.48 and *p* = 0.57, respectively). In total, 25.0%, 36.1%, and 38.9% of the patients were at low, intermediate, and high risk of CHD, respectively, according to FRS, whereas 11.1%, 38.9%, and 50% of the patients were at intermediate, high, and very high risk, respectively, according to the ASCVD score in Group B. We observed that there were no considerable differences regarding the 10-year FRS and FRS subgroups. Furthermore, there were no considerable differences regarding the 10-year ASCVD and ASCVD score subgroups (*p* > 0.05).

## 4. Discussion

Our study showed that among the patients with a planned CACS, 31% and over 50% were at high risk calculated by FRS, and the ASCVD score risk chart with the mean FRS was in the intermediate/high range and the mean ASCVD score was in the high range, with the rate of statin and aspirin use at 13% and 27.5%, respectively. Furthermore, when we looked at the CACS evaluation, we found the presence of subclinical atherosclerosis in 80.7% of the study population, and 16.5% of the individuals were in the high-risk category for CHD, with the rate of statin use at 14% in this group. In a primary prevention screening program in 2018, the U.S. Preventive Services Task Force found that the current evidence was insufficient to assess the balance of benefits and disadvantages of adding CACS to traditional risk assessments for CVD prevention [22]. Moreover, in 2021, the European Society of Cardiology Guidelines on CVD Prevention recommended a class IIb indication for CACS as a risk modifier in CV risk assessment, in addition to traditional risk factors [23]. Additionally, CACS helps guide primary prevention medications. The MESA study showed that participants with CACS > 100 would benefit from aspirin and statin therapy [24]. The 2019 ACC/AHA preventive guideline on assessing CV risk recommends that CACS is a reasonable guide for preventive interventions if decisions are uncertain for adults aged 40–75 years with intermediate ASCVD risk [25]. Recently, Golub et al. summarized the CACS guidelines in atherosclerotic cardiovascular disease risk assessment for applications in preventive therapy. Although there were points of disagreement about the use of aspirin, a common statin treatment threshold was reported as a CAC of over 100 [26]. As this was a retrospective study, we may hypothesize that CACS was planned for our study population, which was free of documented CAD at the time of admission, to improve adherence to primary prevention treatment.

Our results indicate that only 19.3% of the study population was free of subclinical atherosclerosis detected with CACS. As previously mentioned, we included individuals with CACS and retrospectively analyzed the risk categories and medications. We preferred to divide the study population into two to compare the risk status and the medications to draw attention to the need to be cautious when assessing primary prevention. Recent studies showed that a CACS of 0 predicts a low (1.5%–3%) event rate, which may extend to 10 years [13,27]. Mortality was also lower for CACS = 0 individuals who were classified as low-risk or even high-risk according to the FRS [27], and the probability of CV event-free follow-up during the next 12 years was over 90% [8]. Moreover, mortality was not different between diabetics and nondiabetics, and the CV event rate was similar for asymptomatic and symptomatic individuals when the CACS was 0 [28,29,30]. However, the detection of any coronary calcium was associated with adverse coronary events, even in patients with a low risk score [10,31]. The CARDIA study showed that CACS > 0 is common when at least one traditional risk factor is present among the young population [10,11,32]. Moreover, CAC progression can be seen in up to 20% of CACS = 0 individuals within 5 years if any traditional risk factor is present [33,34]. The progression rate is shown to be slow in the first 2 years, with acceleration after 4 years, suggesting a longitudinal follow-up period of 5 years, particularly in patients with cardiovascular risk factors [34]. In our study population, none of the patients had a second CACS within 5 years. However, in the group without subclinical atherosclerosis, the intermediate- and high-risk ratios are high according to the FRS and ASCVD score risk assessments. Furthermore, as half of them are hyperlipidemic, with a low use of statins, it can be predicted that atherosclerosis will begin in the next 5 years if adequate primary prevention measures are not provided in these patients.

Several studies have shown that CACS is a strong predictor of CHD beyond traditional risk factors, and the addition of CACS to standard risk prediction scores resulted in the reclassification of risks in asymptomatic, middle-aged individuals [35,36,37,38]. In the study by Malik et al. [39], higher CACS was associated with a higher ASCVD risk. Moreover, the CARDIA study showed that CACS strongly predicted risk beyond traditional-risk factors among participants aged 32–46 years [32], whereas two other studies showed the predictive value of CACS in elderly patients [40,41]. Our findings were not in accordance with the results of those studies, as we found that 16.5% of the individuals were in the high-risk category for CHD according to the CAC evaluation, and there seemed to be no relationship between CACS-based high-risk and traditional risk prediction models when FRS and ASCVD score were used. This may be because of the following three reasons: First, the FRS is the oldest, most simplified, and most common tool for assessing a 10-year CHD risk calculated with six coronary risk factors: age, sex, total cholesterol, HDL-C, smoking status, and systolic blood pressure [1]. Similarly, the ASCVD score risk calculator uses age, sex, smoking status, total cholesterol, and systolic blood pressure [24]. Therefore, we preferred evaluating the risk with the FRS and ASCVD score risk calculators, which are easily applicable and accessible methods in the primary prevention setting. However, these risk charts exclude some other CHD risk factors, such as diabetes mellitus and family history of CHD. Second, although the CACS population of ≥400 individuals was older, the baseline characteristics were not statistically different between the groups. For both groups, one-third of individuals had a high risk when the FRS calculator was used, and almost half of the individuals were at a very high risk when the ASCVD score calculator was used. To date, only the MESA-CAC score has been validated for combining CACS with the risk calculator model, resulting in a reclassification of risk in 41% of the participants when MESA-CAC was used instead of an ASCVD risk score (23% had a downgraded risk, whereas 18% received an ASCVD risk category upgrade) [38]. Third, the measurement of risk factors once may not reflect the patients’ risk throughout their lifetime.

### Limitations

There are some limitations to our study. This was a retrospective, single-center study with a small sample size of mostly women. At the time of writing, we investigated subclinical atherosclerosis with only CACS and calculated the ASCVD risk with FRS and an ASCVD score risk chart based only on baseline data. Moreover, the new SCORE2 and SCORE2 OP was not used in the date range when the study was conducted. We did not evaluate the progression of CACS when it was 0, or the possible effects of risk factors on this progression. Moreover, we did not evaluate the renewal of prescriptions or patient adherence to optimal primary prevention medication, particularly the use of statins, after CACS.

## 5. Conclusions

Our results support the idea that in primary prevention screening, CACS can be used as a reliable marker of subclinical ASCVD and can help physicians optimize and improve adherence to medical therapy, particularly in higher-risk individuals, even older ones, with aggressive primary prevention strategies, such as aspirin and statins. Moreover, CAC may be a helpful alternative marker in decision-making regarding risk assessment, prognosis, and initiation of primary prevention medications, particularly statins, in patients without subclinical atherosclerosis.

## Figures and Tables

**Table 1:** Baseline demographic and clinical data of the study population.

Variable	Total Study Sample
Age (years), mean ± SD	67.5 ± 10.3
Male, n (%)	75 (34.4)
Symptoms, n (%)	
Non-cardiac chest pain	86, 39.4%
Dyspnea	15, 6.9%
Check-up control	75, 34.4%
Other	42, 19.3%
Diabetes Mellitus, n (%)	83 (38.1)
Hypertension, n (%)	148 (67.9)
Hyperlipidemia, n (%)	104 (47.7)
Smoking status, n (%)	69 (31.7)
Family history of premature CHD, n (%)	53 (24.3)
CACS, mean ± SD (min–max)	258.1 ± 512.9 (0–3726)
10-year FRS, mean ± SD	16.5 ± 9.9
Low risk, n (%)	53 (24.3)
Intermediate risk, n (%)	97 (44.5)
High risk, n (%)	68 (31.2)
10-year ASCVD score risk, mean ± SD	9.8–3.3
Intermediate risk, n (%)	16 (7.3)
High risk, n (%)	85 (38.9)
Very high risk, n (%)	117 (53.7)
Fasting Glucose (mg/dL), mean ± SD	113.9 ± 29.9
HbA1c (%)	6.1 ± 1.0
Total cholesterol (mg/dL), mean ± SD	223.0 ± 47.0
High-density cholesterol (mg/dL), mean ± SD	53.2 ± 15.8
Low-density cholesterol (mg/dL), mean ± SD	140.2 ± 42.9
Triglycerides (mg/dL), mean ± SD	151.9 ± 80.5
Serum creatinine (mg/dL), mean ± SD	0.8 ± 0.1
Baseline Medications:	
Aspirin, n (%)	60 (27.5)
Betablockers, n (%)	76 (34.9)
Angiotensin-converting enzyme inhibitors or receptor blockers, n (%)	118 (54.1)
Calcium channel blockers, n (%)	55 (25.2)
Statins, n (%)	28 (12.8)
Hypoglycemic drugs, n (%)	37 (16.9)

**Table 2:** Demographic and clinical data of the study population, grouped as CACS = 0 vs. CACS > 0.

Variable	Group 1	Group 2	*p*
Number of individuals, n (%)	42 (19.3)	176 (80.7)	
Age (years), mean ± SD	62.9 ± 10.8	68.5 ± 9.9	0.001
Male, n (%)	13 (31)	62 (35.2)	0.60
Diabetes Mellitus, n (%)	12 (28.6)	71 (40)	0.21
Hypertension, n (%)	21 (50)	127 (72)	0.01
Hyperlipidemia, n (%)	18 (42.9)	86 (48.9)	0.49
Smoking status, n (%)	13 (31)	56 (31.8)	1.00
Family history of premature CHD, n (%)	13 (31)	40 (22.7)	0.31
10-year FRS, mean ± SD	13.8 ± 7.0	17.1 ± 10.3	0.04
Low risk, n (%)	13 (31)	40 (22.7)	0.49
Intermediate risk, n (%)	18 (42.9)	79 (44.9)	
High risk, n (%)	11 (26.2)	57 (32.4)	
10-year ASCVD score risk, mean ± SD	9.4 ± 3.7	9.9 ± 3.2	0.38
Intermediate risk, n (%)	6 (14.3)	10 (5.7)	0.11
High risk, n (%)	13 (31.0)	72 (40.9)	
Very high risk, n (%)	23 (54.8)	94 (53.4)	
Fasting Glucose (mg/dL), mean ± SD	113.5 ± 30.3	114.0 ± 29.9	0.89
HbA1c (%)	5.8 ± 0.7	6.2 ± 1.0	0.44
Total cholesterol (mg/dL), mean ± SD	236.7 ± 51.2	220.2 ± 45.6	0.34
High-density cholesterol (mg/dL), mean ± SD	52.1 ± 13.5	53.5 ± 16.3	0.43
Low-density cholesterol (mg/dL), mean ± SD	151.7 ± 44.9	137.4 ± 42.1	0.96
Triglycerides (mg/dL), mean ± SD	164.1 ± 97.3	148.9 ± 76.0	0.33
Serum creatinine (mg/dL), mean ± SD	0.8 ± 0.1	0.8 ± 0.1	0.27
Baseline Medications:			
Aspirin, n (%)	9 (21.4)	51 (29)	0.44
Betablockers, n (%)	10 (23.8)	66 (37.5)	0.10
Angiotensin-converting enzyme inhibitors or receptor blockers, n (%)	13 (31)	105 (59.7)	0.001
Statins, n (%)	6 (14.3)	22 (12.5)	0.79

**Table 3:** Demographic and clinical data of the study population, grouped as CACS < 400 vs. CACS ≥ 400.

Variable	Group A	Group B	*p*
Number of individuals, n (%)	182 (83.5)	36 (16.5)	
Age (years), mean ± SD	66.5 ± 10.5	72.3 ± 7.5	0.002
Male, n (%)	62 (34)	13 (36)	0.81
Diabetes Mellitus, n (%)	68 (37.4)	15 (41.7)	0.70
Hypertension, n (%)	119 (65.4)	29 (80.6)	0.08
Hyperlipidemia, n (%)	84 (46.2)	20 (55.6)	0.36
Smoking status, n (%)	59 (32.4)	10 (27.8)	0.69
Family history of premature CHD, n (%)	43 (23.6)	10 (27.8)	0.67
10-year FRS, mean ± SD	16.3 ± 9.4	17.5 ± 12.1	0.48
Low risk, n (%)	44 (24.2)	9 (25)	0.47
Intermediate risk, n (%)	84 (46.2)	13 (36.1)	
High risk, n (%)	54 (29.7)	14 (38.9)	
10-year ASCVD score risk, mean ± SD	9.8 ± 3.4	9.5 ± 3.2	0.57
Intermediate risk, n (%)	12 (6.6)	4 (11.1)	0.62
High risk, n (%)	71 (39)	14 (38.9)	
Very high risk, n (%)	99 (54.4)	18 (50)	
Fasting Glucose (mg/dL), mean ± SD	112.8 ± 25.1	119.7 ± 47.3	0.01
HbA1c (%)	6.0 ± 0.9	6.4 ± 1.1	0.19
Total cholesterol (mg/dL), mean ± SD	226.5 ± 46.8	207.4 ± 45.8	0.76
High-density cholesterol (mg/dL), mean ± SD	53.1 ± 15.3	54.1 ± 18.4	0.34
Low-density cholesterol (mg/dL), mean ± SD	144.4 ± 42.4	118.9 ± 39.4	0.81
Triglycerides (mg/dL), mean ± SD	151.7 ± 79.9	152.7 ± 84.8	0.51
Serum creatinine (mg/dL), mean ± SD	0.8 ± 0.1	0.9 ± 0.2	0.00
Baseline Medications:			
Aspirin, n (%)	43 (23.6)	17 (47.2)	0.007
Betablockers, n (%)	61 (33.6)	15 (41.7)	0.22
Angiotensin-converting enzyme inhibitors or receptor blockers, n (%)	92 (50.5)	26 (72.2)	0.01
Statins, n (%)	23 (12.6)	5 (13.9)	0.78

## Data Availability

The datasets used and analyzed during the current study are available from the corresponding author on reasonable request.
